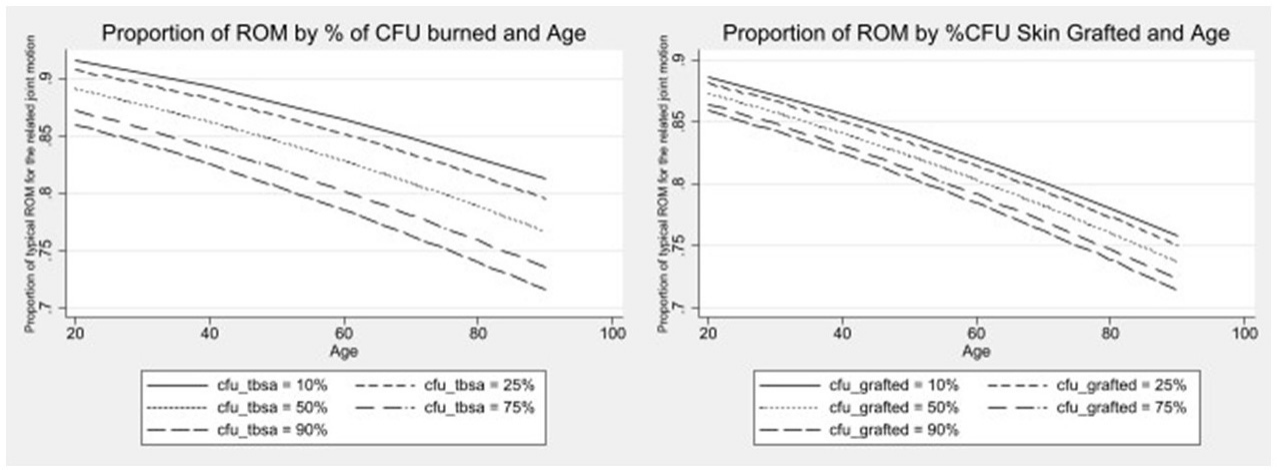# 3 Cutaneous Functional Unit (CFUs) Burn Features Associated with Motion Loss at Hospital Discharge

**DOI:** 10.1093/jbcr/iraf019.003

**Published:** 2025-04-01

**Authors:** Ingrid Parry, Daniel Tancredi, Janice Bell

**Affiliations:** University of California, Davis and Shriners Children’s - Northern California; University of California Davis; University of California Davis

## Abstract

**Introduction:**

Cutaneous Functional Units (CFUs) are regions of skin that elongate to accommodate movement at nearby joints. CFUs are promising for evaluating outcomes after burn injury because they provide patient and injury-specific information about burn location and have functional relevance. However, their current predictive accuracy and clinical utility are limited due to insufficient research quantifying burn characteristics within CFUs. This study evaluated the relationship between multiple acute burn characteristics within CFUs and loss of motion (ROM) in burn survivors at hospital discharge.

**Methods:**

The study used data from a prospective cohort study of adult patients at 13 verified burn centers for secondary analysis. Predictors included five CFU acute burn characteristics: 1) extent (%) of CFU burned, 2) % of CFU skin grafted, 3) depth of burn in the CFU, 4) location of burn in the CFU (proximal or distal to the joint), and 5) % of an adjacent CFU burned. The outcome, proportion ROM, was modeled using fractional regression models with a logit link, yielding effect size estimates analogous to odds ratios: proportion ROM / (1 – proportion ROM). Robust sandwich estimators accounted for clustering of multiple joint motions within patients. Total body surface area (TBSA), body skin grafting, joint motion, sex, age, race, and comorbidities were controlled for in the analysis.

**Results:**

A total of 7463 joint motions from 307 patients were analyzed. Patients were an average of 43.6 (+17.0) years old and had a median TBSA of 8.2% (IQR 4.4-15.7). On average, patients had 24.3 CFUs burned (range 1-82). Proportion ROM decreased for every percentage point increase in the CFU burned (OR=.993; 95%CI.990,.995; p=0.000), grafted (OR=.996; 95%CI.993,.999; p=0.007), and with increasing age (OR=.994; 95%CI.978,.991; p=0.000). Marginal effects are represented in Figure 1. TBSA, body skin grafting, CFU injury depth, and % adjacent CFU burned were not associated with ROM. Sub-analysis of burn location within the CFU showed a significant association with % of burn in the proximal CFU (OR=.993; 95%CI.990,.996; p=0.000), but not the distal CFU.

**Conclusions:**

This study is the first to quantify the relationship between acute burn features within CFUs and ROM outcomes. The proportion of joint motion available at discharge decreases as the extent of burn and grafting in the designated CFU increases, and the effect worsens with age (Figure 1). CFUs provide a granular, functional, and personalized approach to quantifying a burn injury, which could aid in better identifying patients and specific body areas at risk for contractures. The findings of this study can serve as a basis for testing interventions to prevent burn scar contractures and building more efficient models of care.

**Applicability of Research to Practice:**

Improved contracture risk prediction and potential for personalized care

**Funding for the Study:**

N/A